# COVID-19 Outbreak Associated with a 10-Day Motorcycle Rally in a Neighboring State — Minnesota, August–September 2020

**DOI:** 10.15585/mmwr.mm6947e1

**Published:** 2020-11-27

**Authors:** Melanie J. Firestone, Haley Wienkes, Jacob Garfin, Xiong Wang, Kelley Vilen, Kirk E. Smith, Stacy Holzbauer, Matthew Plumb, Kelly Pung, Carlota Medus, Joseph D. Yao, Matthew J. Binnicker, Andrew C. Nelson, Sophia Yohe, Kathryn Como-Sabetti, Kris Ehresmann, Ruth Lynfield, Richard Danila

**Affiliations:** ^1^Minnesota Department of Health; ^2^Epidemic Intelligence Service, CDC; ^3^Division of State and Local Readiness, Center for Preparedness and Response, CDC ^4^Mayo Clinic, Rochester, Minnesota; ^5^Department of Laboratory Medicine and Pathology, University of Minnesota.

*On November 20, 2020, this report was posted online as an *MMWR* Early Release.*

During August 7–16, 2020, a motorcycle rally was held in western South Dakota that attracted approximately 460,000 persons from across the United States to numerous indoor and outdoor events over a 10-day period. During August–September 2020, the Minnesota Department of Health (MDH) investigated a coronavirus disease 2019 (COVID-19) outbreak associated with the rally in Minnesota residents. Fifty-one primary event-associated cases were identified, and 35 secondary or tertiary cases occurred among household, social, and workplace contacts, for a total of 86 cases; four patients were hospitalized, and one died. Approximately one third (34%) of 87 counties in Minnesota had at least one primary, secondary, or tertiary case associated with this rally. Genomic sequencing supported the associations with the motorcycle rally. These findings support current recommendations for mask use, physical distancing, reducing the number of attendees at gatherings, isolation for patients with COVID-19, and quarantine for close contacts to slow the spread of SARS-CoV-2 (*1*). Furthermore, although these findings did not capture the impact of the motorcycle rally on residents of other states, they demonstrate the rationale for consistent mitigation measures across states.

## Investigation and Findings

On August 21, 2020, MDH identified confirmed COVID-19 cases in persons who reported attending the motorcycle rally in the neighboring state of South Dakota. A primary, event-associated case was defined as an illness in a person who reported attending the rally or who traveled to western South Dakota by motorcycle during August 7–16 and who had symptom onset or specimen collection before August 30 (within 14 days after the end of the rally). Reverse transcription–polymerase chain reaction testing for SARS-CoV-2, the virus that causes COVID-19, was used to confirm cases. All confirmed cases among Minnesota residents were reported to MDH. MDH or local public health department staff members interviewed patients with confirmed SARS-CoV-2 infection to identify exposures and persons who might have been in contact with patients during their infectious period (2 days before through 10 days after symptom onset).* To assess exposures, interviews included questions about travel and being in specific settings, such as bars or restaurants, schools, health care facilities, or events or social gatherings in the 14 days before symptom onset. During August–September 2020, MDH and local health department staff members interviewed >80% of patients with a confirmed SARS-CoV-2 infection.

Secondary and tertiary cases were identified from case interview data. Confirmed secondary cases were defined as laboratory-confirmed infections in persons who did not attend the rally but who received SARS-CoV-2–positive test results after having contact with a person who had a primary case during their infectious period. Tertiary cases were laboratory-confirmed cases in persons who had contact with a person who had a secondary case during their infectious period. Likely event-associated secondary cases were confirmed infections in patients who had contact with a person who had symptoms of COVID-19 and had attended the motorcycle rally but who were not tested. Likely event-associated tertiary cases were confirmed infections in patients who had contact with persons who had a likely event-associated secondary case during their infectious period.

To investigate genomic similarity among COVID-19 cases, available SARS-CoV-2 RNA-positive clinical specimens were obtained from clinical laboratories, and whole genome sequencing was conducted at the MDH Public Health Laboratory on 38 specimens using previously described methods (*2*). Phylogenetic relationships, including distinct clustering of viral whole genome sequences, were inferred based on nucleotide differences via IQ-TREE^†^ using general time reversible substitution models (*3*) as a part of the Nextstrain^§^ workflow (*4*). This activity was reviewed by CDC and was conducted consistent with applicable federal law and CDC policy.^¶^

**FIGURE 1:**
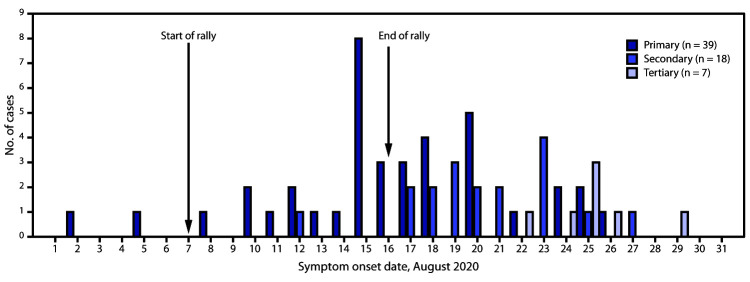
Date of symptom onset among symptomatic patients with primary,* secondary,^†^ and tertiary^§^ COVID-19 (N = 64) associated with a motorcycle rally in a neighboring state — Minnesota, August 2020 **Abbreviation:** COVID-19 = coronavirus disease 2019. * Laboratory-confirmed SARS-CoV-2 infection in a person who attended the motorcycle rally or traveled to western South Dakota by motorcycle during August 7–16 and had symptom onset or specimen collection within 14 days of the end of the rally. ^†^ Laboratory-confirmed infection in a person who had contact with a laboratory-confirmed primary case during the infectious period or with a symptomatic rally attendee who was not tested. ^§^ Laboratory-confirmed infection in a person who had contact with a secondary case or likely event-associated secondary case.

This investigation identified 86 cases, including 51 (59%) primary event-associated cases,** 26 (30%) confirmed secondary and tertiary cases, and nine (10%) likely event-associated secondary or tertiary cases. Four patients were hospitalized, and one died (Table). The median interval between specimen collection and interview was 3 days (range = 1–13 days). Overall, 64 (74%) patients were symptomatic, including 39 (76%) of 51 patients with a primary case and 25 (71%) of 35 patients with secondary and tertiary cases. Among patients with primary cases and symptom onset after the start of the rally, onset dates ranged from August 8 to August 26 (Figure 1). Two patients reported symptom onset before the event and attended the rally during their infectious period. Among primary patients, the median age was 44 years (range = 26–76 years), and 31 (61%) were male. Sixteen (33%) of 48 interviewed patients reported working while infectious, including five who worked at the rally and four who worked in health care after returning from the rally.

**TABLE:** Demographic and clinical characteristics of confirmed* and likely event-associated COVID-19 cases (N = 86) associated with a 10-day motorcycle rally in a neighboring state — Minnesota, August–September 2020

Characteristic	No. of cases (%)		
	Primary event–associated^†^ (n = 51)	Confirmed secondary^§^/tertiary^¶^ (n = 26)	Likely event-associated secondary**/tertiary^††^ (n = 9)
**Demographic**			
**Sex**			
Female	20 (39.2)	16 (61.5)	6 (66.7)
Male	31 (60.8)	10 (38.5)	3 (33.3)
**Age, yrs, median (range)**	44 (26–76)	43 (12–83)	22 (1–51)
**Age group, yrs**			
<18	0 (—)	3 (11.5)	3 (33.3)
18–24	0 (—)	2 (7.7)	2 (22.2)
25–44	26 (51.0)	8 (30.8)	3 (33.3)
45–64	21 (41.2)	10 (38.5)	1 (11.1)
≥65	4 (7.8)	3 (11.5)	0 (—)
**Race/Ethnicity**			
White, NH	43 (84.3)	22 (84.6)	8 (88.9)
More than one race/Other, NH	1 (2.0)	0 (—)	1 (11.1)
Unknown	7 (13.7)	4 (15.4)	0 (—)
**Clinical**			
Symptomatic^§§^	39 (76.5)	19 (73.1)	6 (66.7)
Hospitalized	3 (5.9)	1 (3.8)	0 (—)
ICU admission	1 (2.0)	0 (—)	0 (—)
Died	1 (2.0)	0 (—)	0 (—)
**Close contacts**			
Household	NA	12	3
Social	NA	6	6
Workplace	NA	8	0

**Abbreviations:** COVID-19 = coronavirus disease 2019; ICU = intensive care unit; NA = not applicable; NH = non-Hispanic.

* Receipt of a positive SARS-CoV-2 reverse transcription–polymerase chain reaction test result.

^†^ Laboratory-confirmed SARS-CoV-2 infection in a person who attended the motorcycle rally or traveled to western South Dakota by motorcycle during August 7–16 and had symptom onset or specimen collection within 14 days of the end of the rally.

^§^ Laboratory-confirmed infection in a person who had contact with a primary case during that person’s infectious period.

^¶^ Laboratory-confirmed infection in a person who had contact with a secondary case during that person’s infectious period.

** Laboratory-confirmed infection in a person who had contact with a symptomatic person who attended the rally but was not tested.

^††^ Laboratory-confirmed infection in a person who had contact with a likely secondary case.

^§§^ Symptom status was unknown for three patients with a primary case, three with a secondary/tertiary case, and one with a likely event-associated secondary/tertiary case.

Forty-one (80%) interviewed patients with primary event-associated COVID-19 reported having close contact^††^ with others during their infectious period, with an average of 2.5 close contacts per patient (range = 1–8). Overall, 36 (75%) of 48 interviewed patients with primary event-associated cases reported having close contact with persons in their household while infectious, and 17 (35%) reported having other (social/workplace) close contacts while infectious. Patients reported a total of 59 household contacts (range = 0–4 per patient) and 43 social/workplace contacts (range = 0–6 per patient).

Among the 35 patients with confirmed or likely event-associated secondary/tertiary COVID-19, 25 (71%) were symptomatic, with symptom onset dates during August 12–29 (Table). The median age was 32 years (range = 1–83 years), and 13 (37%) were male. Fifteen (43%) persons with secondary or tertiary COVID-19 were household contacts of a person with a primary or secondary infection, 12 (34%) were social contacts, and eight (23%) were workplace contacts. Secondary transmission from this rally occurred via two workplace outbreaks, one wedding outbreak, and one funeral outbreak. Approximately one third (34%) of Minnesota’s 87 counties had at least one primary, secondary, or tertiary case associated with this rally.

## Whole Genome Sequencing

**FIGURE 2:**
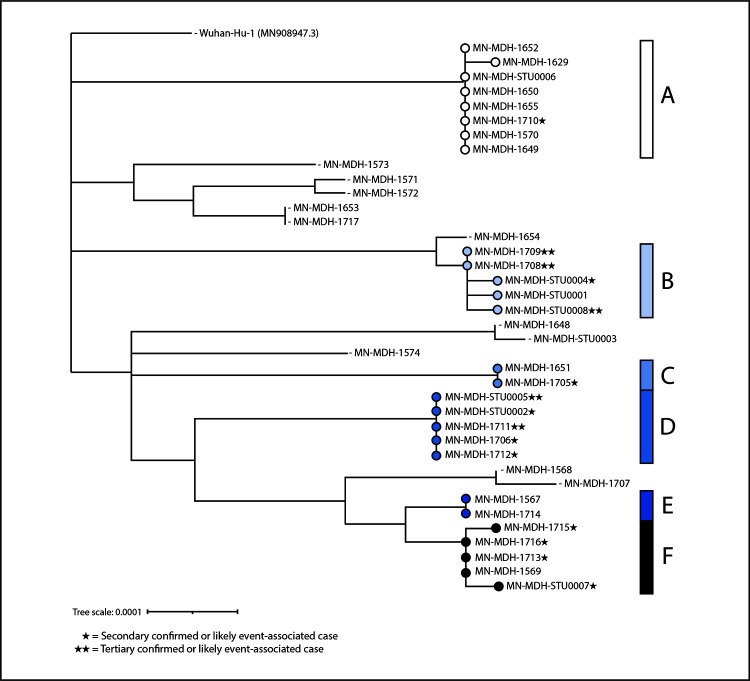
Phylogenetic tree* showing genetic distance between available† SARS-CoV-2 virus specimens collected from South Dakota motorcycle rally attendees and their contacts (N = 38) — Minnesota, August 2020 * This figure was created using Interactive Tree of Life (version 5.7; European Molecular Biology Laboratory). https://itol.embl.de/. ^†^ Genetic divergence based on nucleotide difference is indicated by length of branches in substitutions per site. Available specimens include specimens from clinical labs where specimens could be retrieved and RNA could be extracted.

Specimens were obtained from 52 (60%) patients. Among these, 38 (73%) specimens (23 [61%] from primary and 15 [39%] from secondary and tertiary cases) were successfully sequenced, covering at least 98% of the SARS-CoV-2 genome. Six genetically similar clusters with known epidemiologic links were identified (i.e., cases in patients who were close contacts or who had common exposures at the rally), five of which demonstrated secondary or secondary and tertiary transmission. Cluster A (Figure 2) included genetically similar specimens for seven primary cases and one secondary case (specimen MN-MDH-1710). Among primary cases, specimens were collected from two patients who reported working at the rally, including one who worked at a restaurant. Two other patients in this cluster reported visiting that restaurant. Another patient who attended the rally also reported visiting the same restaurant; this patient was a household contact of the patient with specimen MN-MDH-1710. Cluster B represented a chain of transmission in a workplace setting that included five cases. The secondary case in this cluster (with specimen MN-MDH-STU0004) occurred in a workplace contact of a motorcycle rally attendee (specimen MN-MDH-STU0001) and a social contact of one of the persons with a tertiary case (specimen MN-MDH-STU0008). Another secondary case^§§^ in this cluster was in a workplace contact of the rally attendee and was a household contact of two of the three patients with tertiary cases in this cluster (specimens MN-MDH-1708 and MN-MDH-1709). Cluster C represented secondary transmission from a rally attendee (specimen MN-MDH-1651) to a household contact (specimen MN-MD-1705). Cluster D represented likely event-associated cases of secondary transmission (specimens MN-MDH-STU0002, MN-MDH-1706, and MD-MDH-1712) and tertiary transmission (specimens MN-MDH-STU-0005 and MN-MDH-1711) related to a wedding. The index patient at this wedding reportedly had COVID-19–like symptoms at the wedding after attending the rally but did not receive testing. Cluster E comprised two cases (specimens MN-MDH-1567 and MN-MH-1714) in persons who were household contacts, both of whom attended the rally. Cluster F represents workplace and household contacts (specimens MN-MDH-1715, MN-MDH-1716, MN-MDH-1713, and MN-MDH-STU0007) of the primary patient with specimen MN-MDH-1569. Specimen MN-MDH-1569 was from a musician who performed at the rally and later at another concert with a different band whose members did not attend the rally. Primary event-associated cases with specimens MN-MDH-1571 and MN-MDH-1572 had no known connection to each other or identified common exposure at the rally; however, the reported symptom onset dates and travel dates for these cases were identical. Similarly, primary event-associated cases with specimens MN-MDH-1568 and MN-MDH-1707 had no known epidemiologic link but had identical symptom onset dates. This might indicate a common exposure that was not identified through epidemiologic evidence.

## Public Health Response

On August 5, MDH recommended through media events that motorcycle rally attendees quarantine for 14 days upon return and be tested 5–7 days later even if they were asymptomatic. Attendees and their close contacts with confirmed COVID-19 were instructed to self-isolate. Contacts of patients with confirmed COVID-19 were instructed to quarantine.

## Discussion

Eighty-six Minnesota COVID-19 cases were associated with the South Dakota motorcycle rally; approximately one third of counties in Minnesota reported at least one case epidemiologically linked to this event. These findings highlight the far-reaching effects that gatherings in one area might have on another area. The motorcycle rally was held in a neighboring state that did not have policies regarding event size and mask use, underscoring the implications of policies within and across jurisdictions. The findings suggest that this rally not only had a direct impact on the health of attendees, but also led to subsequent SARS-CoV-2 transmission among household, social, and workplace contacts of rally attendees upon their return to Minnesota. Whole genome sequencing results supported the finding of secondary and tertiary transmission associated with this rally.

The findings in this report are subject to at least three limitations. First, despite in-depth epidemiologic investigation, the findings represent an underestimate of the motorcycle rally’s impact in Minnesota and did not capture the impact within South Dakota or other states. Case interviews were voluntary, and patients could choose not to respond to certain questions. Ten patients reported having close contacts but refused to disclose additional details regarding these contacts. Therefore, it was not possible to identify all contacts of patients who attended the rally. Second, attendees and their contacts might not have been tested for SARS-CoV-2. Two rally attendees indicated that their contacts had COVID-19–like symptoms but did not plan to be tested. As such, the findings underrepresent the number of cases, close contacts, and secondary and tertiary cases. Finally, only 52 specimens were received at the MDH Public Health Laboratory because many testing laboratories do not retain or store specimens long-term. Among these specimens, only 38 were successfully sequenced. The lack of whole genome sequencing data from all cases hindered establishment of complete genetic relatedness for epidemiologic investigation.

A large event in a neighboring state triggered chains of SARS-CoV-2 transmission within Minnesota. Other studies have shown that chains of transmission associated with gatherings are not uncommon within the United States (*5*,*6*). Despite underascertainment of the rally’s full impact in Minnesota and other states, these findings highlight the importance of reducing the number of attendees at gatherings and emphasizing mask use, physical distancing, isolation for patients with COVID-19, and quarantine for close contacts as strategies for reducing the spread of COVID-19. Furthermore, these findings demonstrate the rationale for consistent mitigation measures across states.

**Summary**

**What is already known about this topic?**

Gatherings present an opportunity for rapid spread of COVID-19.

**What is added by this report?**

Following a 10-day motorcycle rally in South Dakota attended by approximately 460,000 persons, 51 confirmed primary event-associated cases, 21 secondary cases, and five tertiary cases were identified in Minnesota residents. An additional nine likely rally-associated secondary or tertiary cases occurred. Four patients were hospitalized, and one died. Genomic sequencing supported the associations with the motorcycle rally.

**What are the implications for public health practice?**

The impact of gatherings as a source of virus transmission underscores the importance of reducing the number of attendees at gatherings, using face masks, and encouraging physical distancing to prevent ongoing transmission of SARS-CoV-2. Furthermore, these findings demonstrate the rationale for consistent mitigation measures across states.

## Footnotes

* The infectious period was estimated to begin 2 days before symptom onset and end 10 days after symptom onset, according to CDC guidance. https://www.cdc.gov/coronavirus/2019-ncov/php/contact-tracing/contact-tracing-plan/investigating-covid-19-case.html.

† http://www.iqtree.org/.

§ https://nextstrain.org/.

¶ 45 C.F.R. part 46, 21 C.F.R. part 56; 42 U.S.C. Sect. 241(d); 5 U.S.C. Sect. 552a; 44 U.S.C. Sect. 3501 et seq.

** One patient reported attending the rally but refused interview. Two additional patients who refused to be interviewed were identified as having attended the rally through secondary case interviews in which other patients reported them as primary event-associated contacts.

†† Close contact was defined as being within 6 feet of a patient with laboratory-confirmed COVID-19 infection for ≥15 minutes. https://www.cdc.gov/coronavirus/2019-ncov/php/contact-tracing/contact-tracing-plan/contact-tracing.html.

§§ Although the specimen from this patient was obtained, sequencing was incomplete.
